# Can passive leg raise predict the response to fluid resuscitation in ED?

**DOI:** 10.1186/s12873-022-00721-6

**Published:** 2022-10-26

**Authors:** MH Elwan, A Roshdy, EM Elsharkawy, SM Eltahan, TJ Coats

**Affiliations:** 1 Emergency Medicine Academic Group, Department of Cardiovascular Sciences, University of Leicester, Level G Jarvis Building RMO, Infirmary Square, Leicester, LE1 5WW UK; 2 Emergency Department, Kettering General Hospital, Kettering, UK; 3 Department of Emergency Medicine, Alexandria University, Alexandria, Egypt; 4 Department of Critical Care Medicine, Alexandria University, Alexandria, Egypt; 5 Intensive Care Unit, North Middlesex University Hospital, London, UK; 6 Department of Cardiology, Alexandria University, Alexandria, Egypt

**Keywords:** Preload responsiveness, Fluid therapy, Haemodynamics, Emergency, Non-invasive monitoring, Bioimpedance

## Abstract

**Objective:**

Passive leg raise (PLR) can be used as a reversible preload challenge to stratify patients according to preload response. We aim to evaluate the accuracy of PLR, monitored by a non-invasive cardiac output monitor in predicting to response to fluid resuscitation in emergency department (ED).

**Methods:**

We recruited adult patients planned to receive a resuscitation fluid bolus. Patients were monitored using a thoracic electrical bioimpedance (TEB) cardiac output monitor (Niccomo, Medis, Germany). A 3-min PLR was carried out before and after fluid infusion. Stroke volume changes (ΔSV) were calculated and a positive response was defined as ≥ 15% increase.

**Results:**

We recruited 39 patients, of which 37 were included into the analysis. The median age was 63 (50–77) years and 19 patients were females. 17 patients (46%) were fluid responders compared to 11 (30%) with positive response to PLR1. ΔSV with PLR1 and fluid bolus showed moderate correlation (r = 0.47, 95% confidence interval, CI 0.17–0.69) and 62% concordance rate. For the prediction of the response to a fluid bolus the PLR test had a sensitivity of 41% (95% CI 22–64) and specificity of 80% (95% CI 58–92) with an area under the curve of 0.59 (95% CI 0.41–0.78). None of the standard parameters showed a better predictive ability compared to PLR.

**Conclusion:**

Using TEB, ΔSV with PLR showed a moderate correlation with fluid bolus, with a limited accuracy to predict fluid responsiveness. The PLR test was a better predictor of fluid responsiveness than the parameters commonly used in emergency care (such as heart rate and blood pressure). These data suggest the potential for a clinical trial in sepsis comparing TEB monitored, PLR directed fluid management with standard care.

## Key messages

**What is already known on this subject?**

- Both over- and under-resuscitation are associated with harm.
- Passive leg raise showed high accuracy in predicting fluid response in ICU and peri-operative settings.
- There is little evidence on the accuracy of passive leg raise in Emergency Department.

**What this study adds?**

- Stroke volume changes with passive leg raise are moderately correlated with fluid bolus with limited accuracy.
- Patients who had a >15% reduction in stroke volume with fluid bolus were accurately predicted by a negative passive leg raise.
- Passive leg raise showed better accuracy than commonly used standard parameters (e.g. heart rate and blood pressure).

## Introduction

Fluid resuscitation is often the first line of treatment for circulatory failure in emergency care, often guided by resuscitation protocols to ensure timely delivery. However, one-size-fits-all protocols have not consistently demonstrated benefit over standard care [1]. The varying needs of resuscitation fluids would result in patients receiving too much and others too little fluids – with the signal of benefit diluted in a heterogeneous group. This suggests that a more individualised approach to shocked patients could be the way forward for optimising resuscitation.

This challenge is most evident in sepsis resuscitation. Protocols commonly recommend a fixed volume of resuscitation fluids followed by escalation of treatment to vasopressors or inotropes [2]. This protocol is recommended for *all* patients, however ‘suspected sepsis’ covers such a wide range of patients that this ‘one size fits all’ approach is unlikely to be correct. In Emergency Department (ED), monitoring the response to treatment relies on standard parameters (e.g. blood pressure and heart rate). These are often insensitive to changes in circulatory volume, cardiac preload or the cardiac output response to fluid resuscitation—and potentially misleading [3].

Standard monitoring parameters are used as proxies for cardiac output (CO) to determine whether patients are responsive following a preload challenge (preload/fluid responsiveness). A positive response means that patients operate on the steep part of Frank-Sterling curve and could tolerate (and potentially benefit from) fluid resuscitation [4]. A negative response indicates operating on the flat part of the curve and may be harmed from fluid resuscitation. While 30 mL/kg of initial sepsis resuscitation is commonly recommended, microvascular damage can happen as early as 9 mL/kg of fluid administered [5]. There is even less room for error with patients at risk of fluid overload in ED.

With no risk of overzealous infusion, Passive leg raise (PLR), a reversible self-fluid challenge, can be used to stratify patients into responders and non-responders [6]. Standard parameters are often insensitive to stroke volume (SV) changes induced by PLR and direct CO monitoring is required [7]. However, the advent of non-invasive cardiac output monitors rendered this approach more feasible in ED [8] PLR seems highly accurate in predicting fluid responsiveness in sedated ventilated patients in intensive care and operating room context [6]. However, there is much less evidence for the accuracy of this approach in ED – where the majority of patients are awake and spontaneously breathing [9].

In this study, we investigated the accuracy of stroke volume response to PLR, monitored by a non-invasive bioimpedance cardiac output monitor, in predicting the stroke volume response to a subsequent fluid bolus in ED.

## Methods

This was a prospective observational study of a convenience sample of adult ED patients (≥ 18 years old) planned to receive at least one resuscitation bolus of IV fluids by the treating team. The study was carried out at Leicester Royal Infirmary, an inner city acute hospital at Leicester, UK. The study was sponsored by the University of Leicester, UK and ethical approvals were obtained from Essex Research Ethics Committee (16/EE/0145).

Exclusion criteria were: clinical condition preventing the performance of a PLR (e.g. trauma), mental health presentations, alcohol intoxication, patients deemed unable to consent due to a pre-existing medical problem (e.g. dementia) and prisoners.

Patients were screened for eligibility by the clinical team or a member of the research team. Recruitment was carried out following a 2-stage consent process. Study procedure was initiated following initial verbal ascent (by patient, personal/professional consultee) following a brief explanation. This was followed by a full informed consent.

On recruitment, we recorded demographic data, presenting complaint, past medical history, and pre-inclusion fluid administered (including pre-hospital). Patients were followed up to record ED diagnosis, final discharge diagnosis, length of stay and mortality data. All biochemical results were recorded.

After the initial consent process standard monitoring was used to record standard physiologic parameters throughout the patients’ stay. From the standard usual care monitoring system the following parameters were recorded at baseline, and at the end of the study:

• Heart rate (HR)
• Respiratory rate
• Temperature
• Systolic blood pressure (SBP)
• Diastolic blood pressure (DBP)
• Oxygen saturation (SpO2)

The TEB monitoring electrodes were applied following manufacturers recommendations—two to each side of the neck and two to each side of the patient’s lower thorax. The study procedure was carried out as follows:

• Patients were monitored for at least 3 min in a 45° head-up position (baseline recording)
• A 3-min PLR test was carried out by trolley manipulation for up to 45° (PLR1)
• Return to baseline position for at least 3 min (baseline 2)
• Fluid bolus administration (typically 500 mL over 15 min)
• PLR was repeated following at least 10 min from fluid bolus end (PLR2)

TEB data were transferred to Microsoft Excel (Microsoft Corporation, United States) and data on CO, SV and HR were extracted. A minute by minute average was calculated for each parameter across the whole time series. To evaluate the overall trend, each parameter was averaged for each minute across the study cohort. To estimate preload responsiveness we considered the haemodynamic variables at seven time periods:

• Baseline1: the minute immediately before leg raise
• PLR1: the middle 1-min of PLR1
• Baseline2: the minute immediately before fluid bolus
• Fluid bolus (FB): immediately following the end of fluid bolus
• Baseline 3: 10 min following the end of fluid bolus
• PLR2: the middle 1-min of the second PLR2`
• Baseline4: at least 2 min following PLR2

Previous studies used a timeframe for assessing the response to PLR ranging 1–10 min [6] A 3-min test was deemed feasible within emergency setting. We chose to estimate fluid responsiveness during the second minute following PLR, to avoid any potential recording noise associated with postural change in conscious patients.

Preload responsiveness was defined as ≥ 15% increase in SV. Accordingly, patients were classified into PLR + /PLR- (responders and non-responders to PLR), and R/NR (responders and non-responders to fluid bolus).

Descriptive data were presented as means with 95% confidence interval (CI), medians with interquartile ranges (IQR) and proportions as appropriate. Data analysis was performed using Graphpad Prism 7 (California, United States). Correlation between the stroke volume changes (∆SV) with PLR1 and FB was calculated using Pearson’s r. Categorical agreement between PLR1 and FB was evaluated using Cohen Kappa. Parametric and non-parametric tests were used as appropriate to evaluate statistical significance of baseline variables between responders and non-responders.

### Patient and public involvement

Patients and public were involved in the design stage through a meeting with the Leicester Cardiovascular Research Review Group. The research protocol was presented to the group and the burden of the intervention was discussed. Feedback from the group was considered during the design and conduct of the study.

## Results

Thirty-nine patients met the inclusion criteria. One patient did not tolerate PLR and another had missing data due to technical failure of data transfer, so 37 patients were included in the analysis. The median age was 63 (IQR 50–77) years and 19 patients (51%) were females. At baseline, the median early warning score (NEWS) was 5 (IQR 3–6) and the majority of patients had a normal blood pressure and a tachycardia. 22 patients (60%) had an infection-related/sepsis as their ED diagnosis (10 in the responder group, and 12 in the non-responder group). Overall, patient characteristics by response to a fluid bolus are shown in Table 1.

**Table 1:** Patient characteristics at baseline

	All *N* = 37	Fluid Responders *N* = 17	Fluid Non-responders *N* = 20	*P* value
Age (years)	63 (50–77)	66 (54–76)	57 (47–78)	ns
Sex				
Female	19 (51%)	9 (53%)	10 (50%)	ns
Male	18 (49%)	8 (47%)	10 (50%)	
Ethnicity				
Caucasian	31 (84%)	14 (82%)	17 (85%)	ns
Asian	4 (11%)	2 (12%)	2 (10%)	
Afro-Caribbean	2 (5%)	1 (6%)	1 (5%)	
Weight (kg)	72 (59–84)	63 (57–78)	79 (64–93)	ns
Height (cm)	174 (159–180)	162 (152–177)	175 (165–180)	ns
BMI	25 (21–30)	25 (22–30)	26 (21–31)	ns
Heart rhythm – n (%)				ns
Sinus	31 (84%)	15 (88%)	16 (80%)	
AF	5 (13%)	1 (6%)	4 (20%)	
Incomplete data	1 (3%)	1 (6%)	0	
Pre-inclusion fluid (ml)	206 ± 363	215 ± 339	197 ± 392	ns
Study fluid				
Volume (mL)	619 ± 224	650 ± 245	593 ± 200	ns
Volume (mL/kg)	9 ± 4	10 ± 4	8 ± 4	ns
Infusion duration (min)	14 ± 7	17 ± 9	12 ± 4	ns
Infusion rate (mL/min)	53 ± 24	50 ± 26	56 ± 22	ns
SBP (mmHg)	119 (100–136)	119 (100–143)	118 (101–136)	ns
DBP (mmHg)	84 (76–99)	72 (65–91)	70 (62–80)	ns
MAP (mmHg)	70 (64–83)	88 (78–109)	84 (75–96)	ns
Heart rate (bpm)	108 (85–125)	109 (92–129)	105 (84–122)	ns
Respiratory rate	22 (19–26)	24 (17–29)	22 (20–24)	ns
Temperature (C)	37 (36.5–38.3)	36.6 (36.2–38.3)	37.3 (36.6–38.2)	ns
O_2_ saturation	97 (94–100)	97 (95–100)	97 (92–99)	ns
New oxygen need – n (%)	8 (22%)	3 (18%)	5 (25%)	ns
NEWS	5 (3–6)	5 (3–7)	4 (3–6)	ns
Base excess	0.9 (-4.3–3.75)	0.55 (-5.53–2.58)	1.2 (-3.3–4.5)	ns
Lactate	1.9 (1.25–3.65)	1.75 (1.15–4.28)	2.0 (1.3–3.6)	ns
Stroke volume (mL)	60 (49–82)	51 (47–56)	72 (60–90)	0.0066
Cardiac output (L/min)	6.1 (4.4–8.1)	5.0 (4.1–6.3)	7.4 (5.7–9.1)	0.0044
Impedance	34 (31–38)	34 (31–38)	33 (29–38)	ns
Signal quality indicator (%)	47 (29–68)	57 (32–70)	44 (27–66)	ns

Data presented as median (interquartile range), mean ± standard deviation or number (percentage) as appropriate. *DBP* Diastolic blood pressure, *MAP* Mean arterial pressure, *ns* non-significant, *SBP* Systolic blood pressure

All patients received a crystalloid fluid bolus with the majority of patients receiving 500 mL with a median of 7.9 mL/kg (actual body weight), IQR 6.1–11. The median time of fluid bolus infusion was 12 min (IQR 9–15.5), with a median fluid infusion rate of 53 mL/min (IQR 33–73).

Thirty-five patients were admitted to a hospital ward (level 0), one patient to intensive care unit (level 3) and another patient to enhanced acute ward (level 1). Patients had a median hospital stay of 5.5 days (IQR 2.25–8), with no significant difference between those who responded to the initial fluid bolus and those who did not (6 vs 5 days, *p* = 0.252). One patient died in hospital.

### Overall haemodynamic changes

**Fig. 1:**
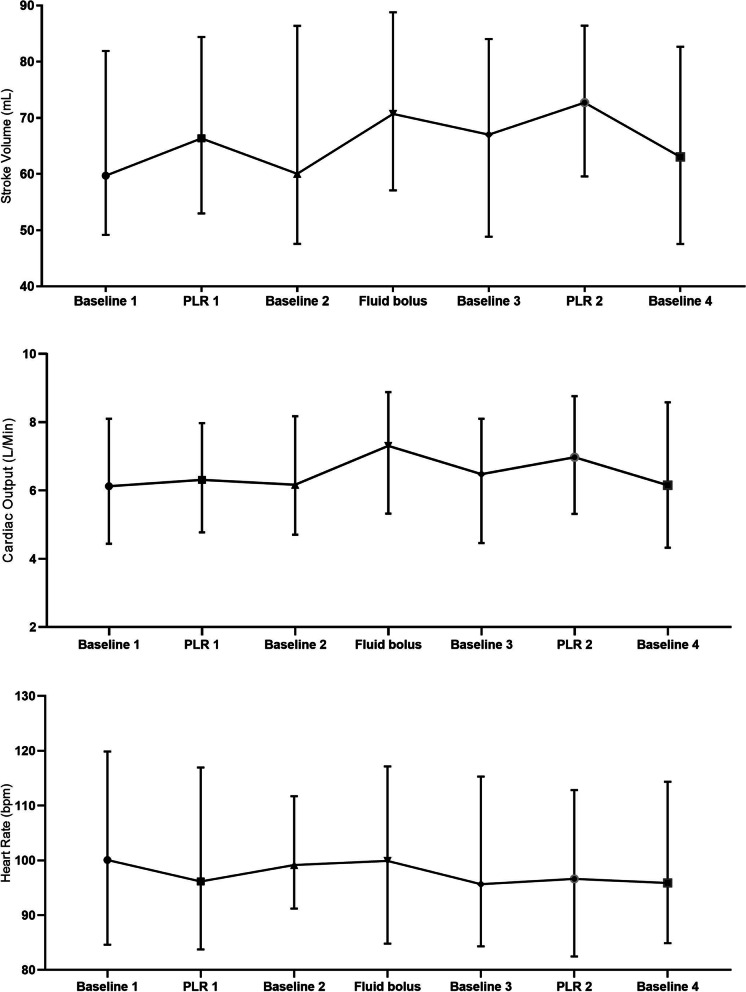
Overall haemodynamic changes. PLR, passive leg raise

TEB monitoring showed that the patients had a median baseline SV of 60 mL which increased to a median of 71 mL (18% increase) immediately following fluid infusion and then fell back to a median of 67 mL at 10 min and was 63 mL (near baseline) by 20 min. The baseline PLR1 Test gave a median increase in SV of 10%, with the PLR2 test (after fluid infusion) giving the same result (Fig. 1). The median CO showed a similar pattern, albeit with less pronounced changes during the PLR tests. Heart rate data did not show any observable change with PLR or fluid bolus.

### Haemodynamic changes with baseline PLR

Eleven patients (30%) showed a positive SV response (≥ 15%) to the baseline PLR test. with a median SV increase of 30% following the PLR manoeuvre, which then rapidly fell back to baseline. In these patients, the median SV increased following fluid bolus by 22% with partial resolution at 10 min after infusion. When the PLR test was repeated after fluid infusion in this patient group there was a median increase of SV by 18% again rapidly reversed after the manoeuvre.

**Fig. 2:**
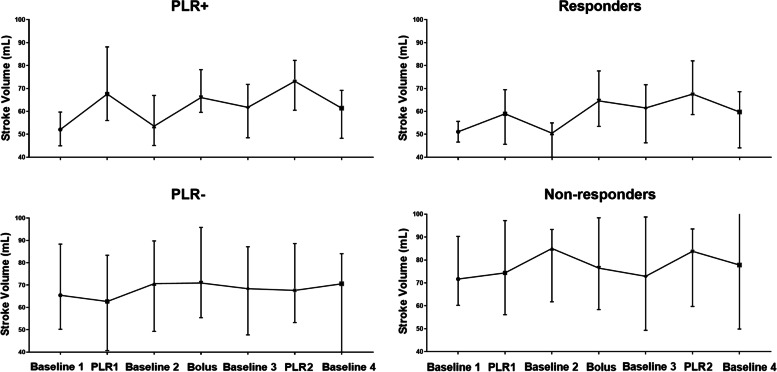
Stroke volume changes classified by responsiveness to passive leg raise (PLR  + ve/-ve) and by fluid responsiveness (responders/non-responders)

In the patients with a negative baseline PLR test there was no change in median SV with the fluid bolus and again no change in SV with the PLR test after fluid infusion (Fig. 2).

### Haemodynamic changes with fluid bolus

#### Fluid responders

Seventeen patients (46%) responded to the fluid bolus (≥ 15% SV change). In these ‘fluid responders’ the median SV increase following fluid bolus was 28%, with partial resolution to 22%, 10 min later. This group of patients had a median SV increase of 16% during the baseline PLR test, with a rapid fall in SV back to the baseline after the test. When the PLR test was repeated after the fluid bolus there was a median SV increase of 10%, which again rapidly reversed after the test.

#### Fluid non-responders

In the patients who did not have a change in SV (median –9% in response to the fluid bolus, there was only a small 3% rise in SV during the baseline PLR test, with a larger (but still borderline for a ‘response’) increase in SV (15%)in response to the PLR test performed after the fluid bolus (Fig. 2).

### Relationship between PLR1 and FB haemodynamic changes

**Fig. 3:**
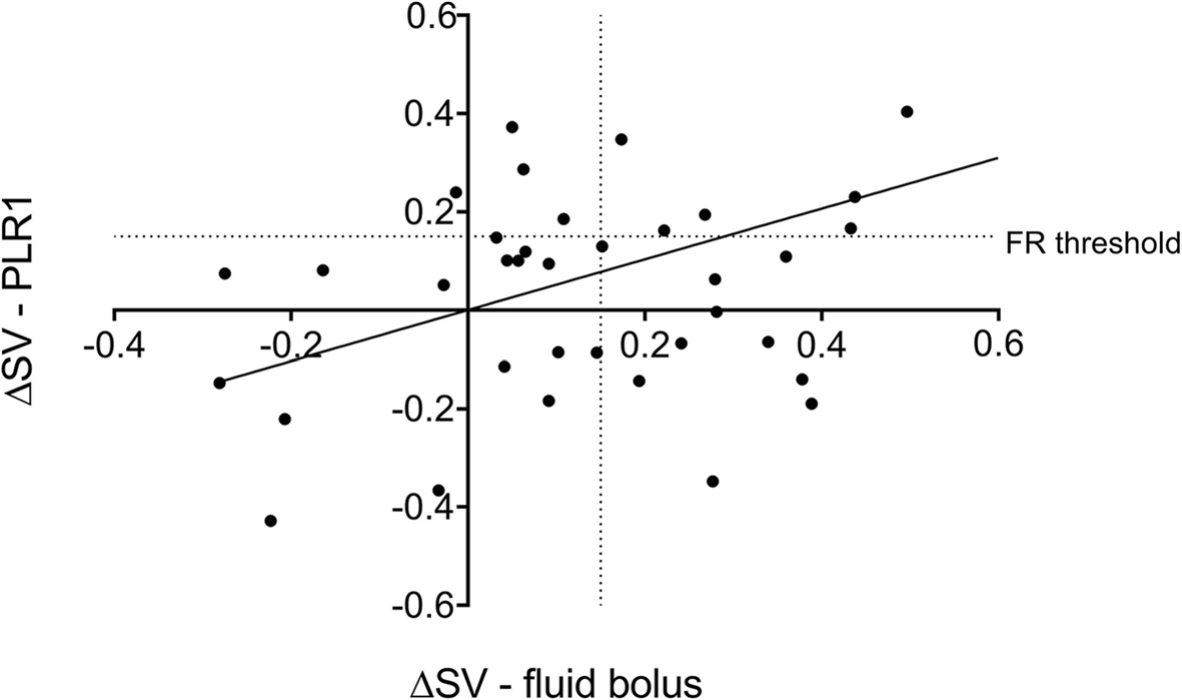
Correlation between stroke volume changes (ΔSV) with fluid bolus and passive leg raise 1 (PLR1). One point is outside the axis limit. FR, fluid responsiveness

Change in SV (ΔSV) during the baseline PLR1 test showed a moderate correlation with ΔSV after fluid bolus—Pearson r of 0.47 (95% CI 0.17–0.69) and 62% concordance (23 patients), Fig. 3. It is noticeable that all of the patients who had a large adverse effect (decrease in SV) from the fluid bolus (those to the left of the ‘y’ axis) were “non-responders” to the baseline PLR test (are below the 15% horizontal dotted threshold for fluid responsiveness). Conversely, there were a number of patients who had a large increase in SV following the fluid bolus (those to the right of the ‘y’ axis), but were “non-responders” to the Baseline PLR test (below the dotted FR threshold).

To predict fluid responsiveness (ΔSV ≥ 15% with fluid bolus), the Baseline PLR test had a sensitivity of 41% (95% CI 22–64) and specificity of 80% (95% CI 58–92). This gave a positive predictive value of 64% (95% CI 35–85) and a negative predictive value of 62% (95% CI 43–78). The positive likelihood ratio was 2.06 (95% CI 0.72–5.85), while the negative likelihood ratio was 0.74 (95% CI 0.47–1.16). PLR1 had an area under the curve of 0.59 (95% CI 0.41–0.78) – P value 0.33.

We analysed the degree of concordance between PLR1 and FB using different thresholds (0–15%) to define ‘positive response’ to both the baseline PLR test and the fluid bolus. The highest concordance (62%) was achieved using the 15% threshold for both PLR1 and FB (Table 2).

**Table 2:** Concordance between Baseline PLR test and fluid bolus using different positive response thresholds

	> 0% FB	≥ 5% FB	≥ 10% FB	≥ 15% FB
> 0% Baseline PLR	60%	57%	46%	49%
≥ 5% Baseline PLR	60%	57%	46%	49%
≥ 10% Baseline PLR	60%	60%	54%	58%
≥ 15% Baseline PLR	46%	54%	58%	62%

### Accuracy of baseline parameters to predict fluid responsiveness

**Fig. 4:**
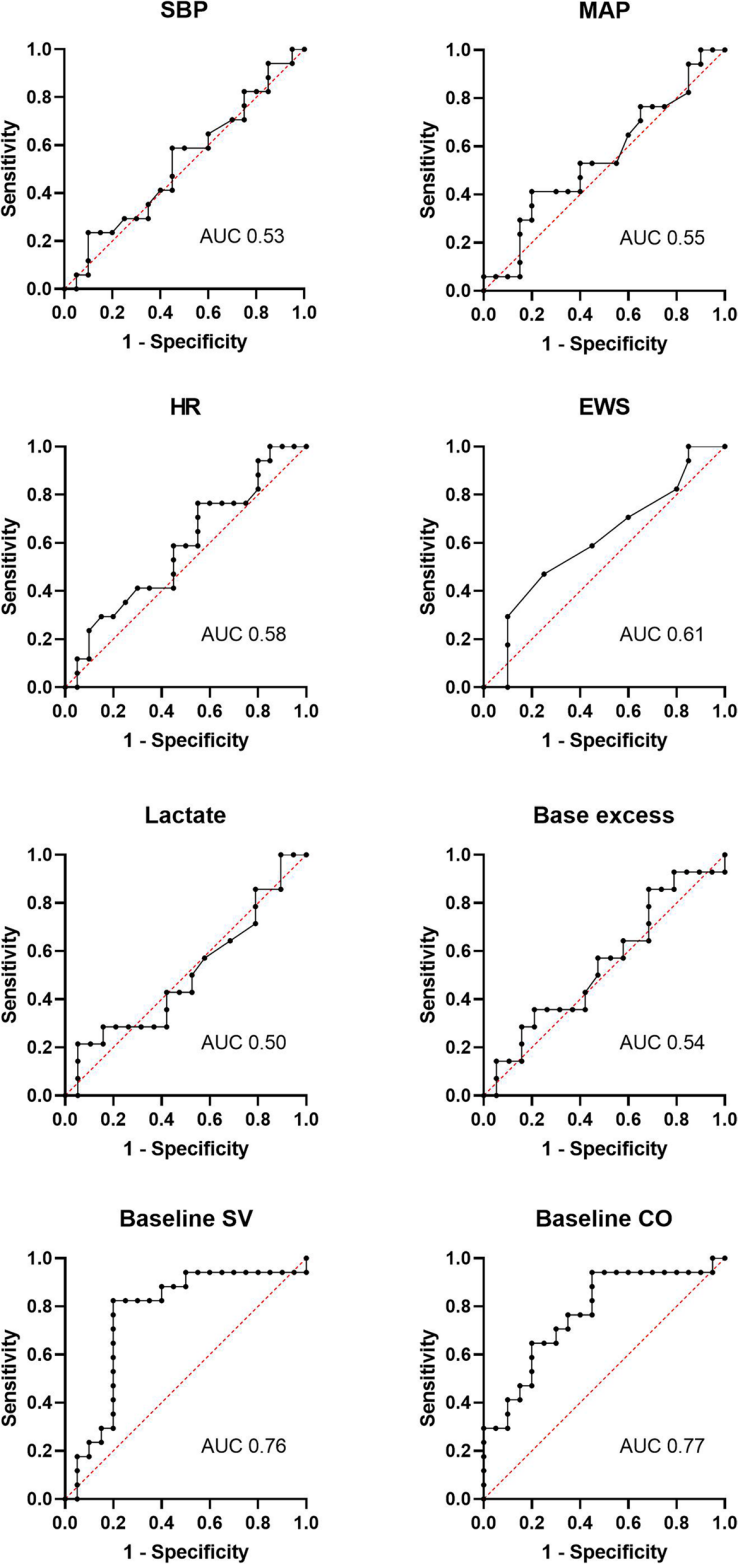
Receiver operator curve for the accuracy of baseline parameters in predicting fluid responsiveness. CO, cardiac output; EWS, early warning score; HR, heart rate; MAP, mean arterial pressure; SV, stroke volume; SBP, systolic blood pressure

The parameters conventionally used in emergency care to assess volume status and determine fluid management, such as pulse rate and blood pressure, showed poor performance in predicting fluid responsiveness (Fig. 4). The AUC for these parameters are shown in Fig. 4, showing lower predictive value than baseline SV and CO – which seemed to be better predictors of response (Fig. 4).

## Discussion

In this work, we found that SV changes with PLR test were moderately correlated with SV changes following a fluid bolus. In a sample of 37 patients, the test showed a reasonable specificity of 80%, but a poor sensitivity of 41%. This suggests that a patient with a positive Baseline PLR test is likely to respond to a fluid bolus, but that a patient with a negative baseline test may or may not respond.

The majority of our patient cohort were elderly and not critically unwell – which is typical of a UK ED sepsis population. In the UK, sepsis protocols use EWS along with suspected infection to trigger a fluid challenge as part of the ‘sepsis six’ bundle, so it is normal for this treatment to be given to patients before they become critically unwell.

Conceptually, preload challenge is assumed to increase venous return, then increasing right ventricular diastolic volume, with a subsequent increase in left ventricular SV (preload responsiveness). Fluid challenge is the gold standard for assessing preload responsiveness, however variation in the challenge technique (volume and rate) may affect the outcome. A meta-analysis by Toscani et al. showed heterogenicity in technique and that an infusion time > 30 min was associated with a lower proportion of responsiveness [10]. A systematic review of ICU and ED fluid challenges found that the infusion time was variable (median of 30 min, range 3 to 60) [11]. Similar large variation in practice was seen in the FENICE study, with a median of 24 min [12]. The effect of variation in the dose of fluid challenge on fluid responsiveness was examined by Aya et al. with a conclusion that a dose of at least 4 mL/kg should be used [13].

Stroke volume change (ΔSV) in both PLR tests (before and after fluid bolus) was positive in 30% of patients, which is within the range reported in previous literature [9]. However a higher proportion of patients were responsiveness (ΔSV) to the fluid bolus (46%). This higher preload response to FB compared to PLR has previously been seen in both mechanically ventilated and spontaneously breathing patients [14, 15]. There are several possible explanations for this observation. The fluid bolus may give a greater preload challenge than PLR (which only cause around 300 mL of fluid shift—which may be even less in the dehydrated patient). Undertaking PLR in awake patients may also be associated with neural responses (e.g. vagal stimulation) which may influence the ΔSV [16].

Stroke volume changes (ΔSV) with baseline PLR showed a moderate correlation with ΔSV following fluid bolus with 62% positive concordance (Fig. 4). Past research on fluid challenge has concentrated on identifying the patients who need fluid resuscitation, however it can be seen that all patients who had a large negative response to FB (> 15% decrease in SV) were predicted by a negative Baseline PLR test. Future studies might use the PLR test to identify those patients who might be adversely affected by a fluid bolus.

While PLR had limited diagnostic performance in predicting FR, none of the standard monitoring parameters showed better performance. Notably, Baseline SV and CO showed a higher predictive ability than any of the currently used parameters (such as pulse rate and blood pressure), which is consistent with our volunteer results [16]. This generates an interesting hypothesis of whether baseline SV (without the PLR) could add value to the assessment of volume status in ED.

In our experiment, PLR was able to stratify patients into two different groups according to fluid responsiveness. However, it seemed to be testing a cardiovascular response that was related to, but different from the response to a fluid bolus. Using a diagnostic threshold of an increase in SV > 15% failed to show adequate accuracy for PLR alone as a definitive diagnostic test to predict fluid responsiveness, however as it performed better than any of the currently used parameters.

### Limitations

Our study included a convenience sample of ED patients and may be liable for selection bias. A single operator (ME) carried out the procedure so we cannot present any data on inter-rater reliability. Operator was not blinded to SV changes during PLR and fluid bolus. However, this is less likely to have introduced bias as the analysis was carried out using a standardised code blinded to individual SV changes.

In our study, data analysis did not happen in real-time and was based on summary numbers over time windows. This is not strictly comparable to the clinical situation where the clinician at the bedside is able to look at the continuous SV trace. This gives additional information about the reliability of the data, such as the stability of signal and the degree of artefacts observed (similar to the interpretation of pulse oximetry at the bedside). This additional information (which could not be used in this study without introducing observer bias) might change the utility of SV monitoring in real life.

A potential limitation of the study is that, as expected in older people, five patients had atrial fibrillation. The TEB monitoring technique is known to be less accurate in these patients, however they were included in the study to ensure that the results were generalisable to a normal ED sepsis population. A further limitation is that compared with previous ICU studies the ED patients had relatively little fluid before the PLR test – this may have reduced the fluid shift caused by the PLR, and given a false negative result. However, most ED patients have little fluid resuscitation on presentation, so the study population is representative of normal practice.

Another caveat is the specific time windows for evaluating a dynamic process (e.g. evaluating fluid responsiveness in a fixed time window immediately following fluid infusion). While this method has some evidence base, there is a disadvantage in artificially imposing time windows [17]. In real life a clinician would continuously inspect the pattern of change over time (rather than forcing a judgment within a specific time)—a complex process of clinical judgement which is difficult to replicate mathematically. Another potential limitation is forcing patients into binary responder/non-responder groups using the artificial 15% change. There are probably degrees of response, for example our data suggest that negative responder (> -15% ΔSV), positive responder (> + 15% ΔSV) and intermediate response may be useful future categories.

## Conclusion

The PLR test showed a moderate correlation with SV changes following a subsequent fluid bolus, with a limited accuracy to predict fluid responsiveness. Based on our data, the low test sensitivity, does not allow for a safe prediction of over-resuscitation. However, from our observations, a group of patients who have an adverse response to the conventional sepsis management protocol might be predicted by a negative SV change on baseline PLR test. The PLR test was a better predictor of fluid responsiveness than the parameters commonly used in emergency care (such as heart rate and blood pressure) which had poor accuracy. In the context of what is already known about cardiac monitoring and fluid challenges, these data suggest the potential for a clinical trial in sepsis comparing TEB monitored, PLR directed fluid management with standard care.

## Data Availability

Please contact author for data requests.
